# Comparing models of learning and relearning in large-scale cognitive training data sets

**DOI:** 10.1038/s41539-022-00142-x

**Published:** 2022-10-04

**Authors:** Aakriti Kumar, Aaron S. Benjamin, Andrew Heathcote, Mark Steyvers

**Affiliations:** 1 University of California, Irvine, CA USA; 2 University of Illinois at Urbana-Champaign, Champaign, IL USA; 3 University of Newcastle, Newcastle, Australia

**Keywords:** Human behaviour, Human behaviour

## Abstract

Practice in real-world settings exhibits many idiosyncracies of scheduling and duration that can only be roughly approximated by laboratory research. Here we investigate 39,157 individuals’ performance on two cognitive games on the Lumosity platform over a span of 5 years. The large-scale nature of the data allows us to observe highly varied lengths of uncontrolled interruptions to practice and offers a unique view of learning in naturalistic settings. We enlist a suite of models that grow in the complexity of the mechanisms they postulate and conclude that long-term naturalistic learning is best described with a combination of long-term skill and task-set preparedness. We focus additionally on the nature and speed of relearning after breaks in practice and conclude that those components must operate interactively to produce the rapid relearning that is evident even at exceptionally long delays (over 2 years). Naturalistic learning over long time spans provides a strong test for the robustness of theoretical accounts of learning, and should be more broadly used in the learning sciences.

## Introduction

Acquiring substantial skills or knowledge takes consistent and ongoing practice. Real-world skill-building and learning is an activity that is distributed over time in complicated ways. Some of the earliest systematic studies of learning revealed that *spacing* practice events in time enhances long-term performance^1,2^; those results have been confirmed hundreds of times, including in large-scale studies^3^, meta-analysis^4^, and in translational environments like a school classroom^5^.

Yet laboratory studies of learning have important limitations^6,7^. The typical cognitive experiment is careful to control for the many factors that complicate learning, including variability and gaps in regularly scheduled spacing. This characteristic makes such experiments incomplete models for understanding the full complexity of learning schedules. A student is rarely on a systematic schedule when studying for an exam. They determine when they study and for how long they study. Their learning schedule must accord with other obligations, and will be influenced by circadian variables that affect sleep and attention. All of this is to say a comprehensive theory of spacing and learning should be accommodating of schedules that are less regimented than the ones implemented in the controlled conditions of the laboratory^8^. Here we examine learning and retention in an online-training platform, Lumosity, to capture real-world constraints on learning schedules and also to examine learning over extended time spans that are rarely considered by lab experiments.

The goal of this article is twofold. First, we use the performance data within two games in Lumosity to characterize aspects of learning that appear in naturalistic learning data but are mostly absent in laboratory studies of learning. Second, we introduce a hierarchy of simple models of learning and demonstrate through applications to this data set that a successful model of learning must include mechanisms for skill learning, task-set preparedness, and forgetting to apply to the time scales relevant for the variety of conditions in which learning takes place. Most importantly, we identify a critical component that such models must possess in order to fully account for relearning over multiple sessions: an interaction between long-term skill learning and short-term task-set preparedness.

**Fig. 1:**
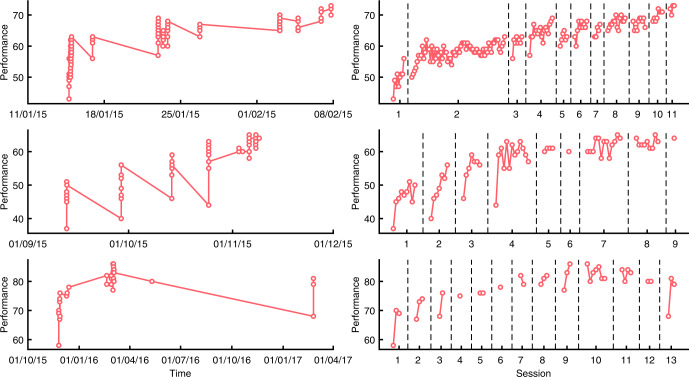
Example learning curves for three individuals in the Lumosity data set (rows). In the left panels, time is expressed as the date and time stamp at which each gameplay occurred. Circle markers indicate individual gameplays. Right panels show time expressed as session and gameplay within session. Performance is assessed by the number of correct decisions within one gameplay (see Methods for details).

Ceding control over learning to the learner introduces novel aspects to performance data and poses challenges to typical theoretical approaches. Some of these characteristics can be seen in the learning functions shown in the left panels of Fig. 1, which are drawn from the data set we introduce here. Unlike traditional learning curves, it is clear that there are massive gains within short periods of time (a single session of practice) and also extreme gaps in practice that offset those gains. Rendering these plots in terms of gameplays rather than chronological time, as is done in the right panels, reveals more traditionally appreciated aspects of learning curves—that performance rises with practice, and that it does so in a negatively accelerating manner—but obscures the true patterns of learning and forgetting apparent in the left panels.

**Fig. 2:**
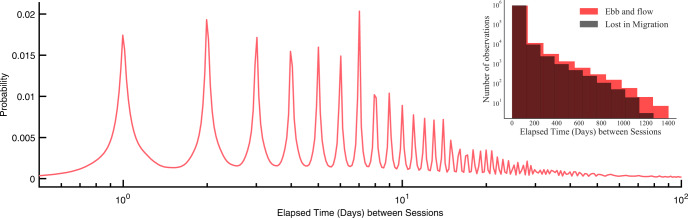
Distribution of elapsed time between sessions in the Lumosity training data. The outer panel shows the elapsed time in days on a common logarithm scale. Peaks correspond to multiples of day intervals. The inner panel shows the distribution of elapsed times in our sub-sample of two cognitive games.

Because practice is under the control of the learner and dependent on their idiosyncratic schedules, group effects reveal expected but important regularities in distributions of practice. Figure 2 shows the distribution of elapsed time between consecutive sessions in the data set used here. The peaks at multiples of 24 h show that individuals practice at regular intervals spaced days apart. A weekly cycle is also evident in the distinct peaks at 7 day intervals. Finally, the relatively low probability for the troughs between the peaks indicates that users practice at roughly consistent times of day. Users space practice sessions with great variability, often on the order of days, but sometimes over weeks and even months. A theory of learning that cannot accommodate learning that takes place over these wide time scales that characterize real-world learning must be considered incomplete.

Learners schedule practice systematically, with an eye towards making efficient use of their limited time. Research on metacognitive control over learning reveals that self-control is highly beneficial for rote verbal learning^6,9,10^, spatial learning^11^, motor learning^12^, and category and causal learning^13^. Naturalistic data sets contain valuable information about self-scheduling behaviour and could be compared to experimental studies with control groups to assess the efficacy of naturalistic metacognitive behaviour.

Long-term learning functions reveal two timescales of learning^14^. Within each session, gains in performance are rapid: note, for example, that the three subjects in Fig. 1 exhibit gains within the first session of practice that account for 50-70% of asymptotic performance. Across sessions, gains are slow but steady; these can be most easily seen in the right panels. Finally, between sessions, losses are evident but rapidly recovered. These three features are the key desiderata of models of naturalistic long-term learning.

An important point of contact between naturalistic learning data and the traditional cognitive literature on learning is the investigation of schedules that vary the spacing across multiple learning events. Schedules that *expand* the intervals (or the difficulty) between successive learning events^15,16^ are better for long-term retention than schedules in which events are equally spaced or in which spacing is reduced over time. This result is consistent with the idea that the rate of forgetting following each successive learning event is progressively reduced. This idea has major implications for theories of learning, because it is incompatible with the idea that forgetting and learning *independently* contribute to performance. We will carefully consider here which components of learning persevere through extended droughts of practice and which are lost.

When systematic rest periods between sessions of practice are included in an investigation of learning, two important phenomena arise. First, performance drops over the interval of rest. Though this result is intuitive, it reminds us of the important fact that forgetting occurs over the course of learning, and that eventual performance is the outcome of a balance between ongoing learning and ongoing forgetting. Second, and less intuitively, performance rises very rapidly during relearning after short gaps—so rapidly, in fact, that many models of learning are easily rejected once this point is seriously considered. It is this aspect of real-world learning that is probative with respect to the underlying dynamics relating learning and forgetting, and that is a central target of our analysis here.

Historically, the drop in performance following rest, followed by rapid gains in performance during relearning, has been known as the *warm-up decrement*, a term that reflects the view that the dip in performance owes principally to factors that moderate the *expression* of skill, rather than factors that reflect skill directly^17,18^. Such factors might include attentional state, preparedness, and general familiarization with the task and the interface^19^. We will refer to these factors collectively as “task-set preparedness”. This distinction between true long-term skill at a task and the preparatory state of the subject plays a key role in the theoretical position that we develop here.

Accommodating models of learning with the warm-up decrement requires a explicit recognition of the fact that all learning functions reflect a combination of both growth and decay. Practice enhances skill and improves performance, but forgetting during periods of rest offsets those gains. This juxtaposition of learning and forgetting is the central piece of an important model proposed by^20^ and its descendants (e.g., ref. ^21^), in which performance on a task is determined by the sum of individual learning events, each of which experiences power-law forgetting with time. That model accounts well for the general course of learning and for the cost of intervals in which practice is ceased^22^. A related theory was suggested by ref. ^23^, in which a latent variable (*storage strength*) mediates the magnitude of gains from practice and losses from forgetting depend on *retrieval strength*, the variable that determines manifest performance. Like the model of ref. ^20^, the overall learning function is a balance of learning and forgetting. These views are of a piece with theories in motor learning that emphasize learning over multiple time scales, some of which lead to more permanent learning than others^14^.

**Fig. 3:**
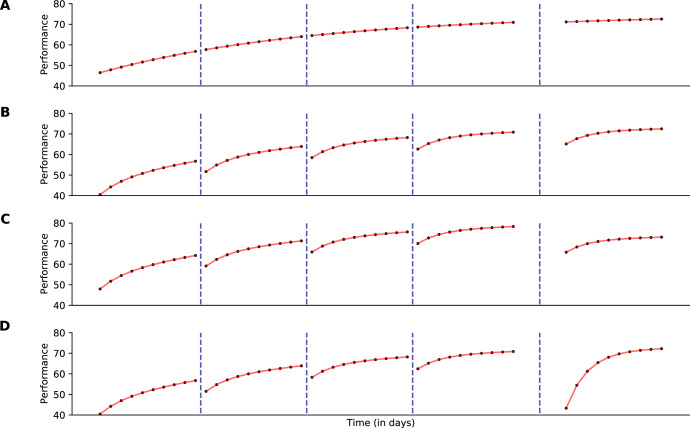
Model predictions for a hypothetical experiment with five sessions of practice and ten games in each session. Dashed lines separate sessions. All sessions are spaced 1 day apart except for the final session, which occurs 300 days after the previous session. Panel **A** shows predictions for *M*_1_, panel **B** for *M*_2_, panel **C** for *M*_3_, and panel **D** for *M*_4_. The parameters used to generate model predictions are A = 75, *U* = 30, *λ* = . 05, *γ* = 0.008, *β* = 0.5, *δ* = 0.25, and *τ* = 0.2.

A model of learning that does not incorporate forgetting during periods of inactivity makes predictions about long-term learning that are obviously but meaningfully wrong. An example is shown in the top panel of Fig. 3, where learning reflects practice but not rest. Taking this model as a starting point, what is lacking are mechanisms that reduce performance during rest—forgetting—and rapidly regain performance during the initial trials of each practice session.

More realistic performance is evident in a model, shown in the second row, that allows for two timescales for learning^14^. It yields plausible effects of rest—during which skill is maintained but task-set preparedness is lost—and greater verisimilitude with the sample learning functions shown in Fig. 1. Yet this model does not effectively handle the fact that some gaps in practice are longer than others: once task-set preparedness is lost, no additional losses in performance are possible.

A third model, shown in the penultimate row, introduces genuine forgetting across sessions to the two timescales of learning. In that model, forgetting balances against the two timescales of learning, but is agnostic with respect to the nature of the information that is forgotten. Importantly, the loss is a function of the actual delay from one session of practice to the next. Longer breaks reduce performance more dramatically, but relearning proceeds at the same pace regardless of overall skill.

The final model, shown in the bottom panel, includes forgetting, learning over two time scales and, critically, an interactive component between those two components of learning. In that model, task-set preparedness is lost between sessions but is also more rapidly regained with each additional practice session. The diagnostic prediction of this model is the increase in the rate of the learning function across practice sessions. This approach is represented well by the models of^22–24^, which feature interactivity between competing processes. This interactivity has also been empirically documented within the empirical literature on relearning and noted as a characteristic aspect of learning schedules that contain periods of learning and forgetting^25,26^.

Here we formulate the four models used to generate the hypothetical data shown in Fig. 3. *M*_1_ is a baseline learning model that is not designed to capture any within-session learning or forgetting over long delays between sessions. *M*_2_ includes two timescales for learning but no explicit forgetting. *M*_3_ adds to *M*_2_ forgetting that varies with delay. Finally, *M*_4_ adds to *M*_2_ a degree of *interactivity* between the two learning components; this interactivity yields both forgetting and relearning. In the Supplementary Materials, we review and describe the predictive capacity of the Predictive Performance Equation (refs. ^21,24^), a benchmark model for understanding the effects of spacing and forgetting on learning. This model was not designed to handle the extreme timescales evident in the Lumosity data, and so we do not directly compare it with the more rudimentary models presented here.

The baseline learning model (*M*_1_) is based on standard learning functions designed to account for the overall effect of practice based on exponential and power law learning functions^27–29^. We consider a simple three-parameter exponential learning model that only accounts for the overall effect of practice, regardless of session:

1 $${y}_{j}=A-U{e}^{-\lambda j}$$

In this model, performance *y* is expressed as a function of *j*, the overall number of games played. The learning function has three parameters: learning rate *λ*, gain parameter *U* and asymptote *A*. We will assume a parameterization in which trials are zero-indexed: for the first game, *j* = 0. With this parameterization, performance starts at baseline *A* − *U* and after extended practice, approaches the asymptote *A*.

The two-timescale learning model (*M*_2_) is a model intended to implement the approach described by^30^, in which performance is based on two learning processes that operate on different time scales. The “slow” learning process captures the improvement in overall skill whereas the second “fast” learning process captures the changes in the attentional state, task knowledge and general level of preparedness, all of which increase rapidly within a session and drop rapidly between sessions. The model incorporates within-session learning as an additive combination of these two learning processes:

2 $${y}_{j}=A-U({e}^{-\lambda j}+\tau {e}^{-\beta k})$$

In this model, performance *y* is expressed as a function of the overall number of games played (*j*) and the game play number within the session associated with the *jth* game, *k*. The within-session game play number (*k*) resets at the start of every new session and is zero-indexed (i.e, for the first game in every session, *k* = 0). Similar to the baseline model *M*_1_, this parameterization leads to baseline performance *A* − *U* on the first game play and after extended practice, performance approaches the asymptote *A*. The model has two learning rate parameters: *λ*, for the slow learning process, and *β* for the fast within-session learning. The parameter *τ* determines the size of the within-session learning effect relative to the overall effect of learning across gameplays. When this parameter is set to 0, this model reduces to *M*_1_. Model *M*_2_ allows for rudimentary forgetting via the loss of task-set preparedness between sessions. However, this aspect of the two-timescale model is incompatible with a core characteristic of forgetting—namely, that it grows with time. The two-timescale learning model with forgetting (*M*_3_) adds to the two components of learning an explicit forgetting component:

3 $${y}_{j}=A-U({e}^{-\lambda j}+\tau {e}^{-\beta k}-\delta {e}^{-\gamma {t}_{j}})$$

As in the previous models, these components are combined additively. Parameters are the same as in *M*_2_, with the addition of an additional weighting factor (*δ*) and rate (*γ*) for the forgetting component where *t*_*j*_ is the elapsed time between the sessions associated with game plays *j* and *j* − 1. While it is possible that learners may have different forgetting patterns, not all participants have enough data to predict a stable individual specific gamma. Hence we assume *γ* to be shared across participants.

The interactive model (*M*_4_) is designed in the spirit of models proposed by refs. ^21–23,31^. Performance reflects the contribution of learning over multiple time scales, but the nature of forgetting reflects a distinction between the permanence of skill and the impermanence of task-set preparedness. Like the ability to ride a bike, acquired skill is maintained permanently, without loss. Task-set preparedness, in contrast, is labile, and varies with changes in context. Forgetting and relearning of that information is thus governed in part by the delay between sessions of practice. *M*_4_ allows forgetting to increase with delay between sessions; unlike *M*_3_, that contribution is not additive, but interactive:

4 $${y}_{j}=A-U({e}^{-\lambda j}+(\tau +(1-\tau )L({t}_{j})){e}^{-\beta k})$$

As in the previous models, the term *e*^−*λj*^ indexes the slow learning process and is based exclusively on the total number of games played (*j*). Improvements in performance due to slow learning are not subject to forgetting. The term *e*^−*βk*^ captures the fast learning process that represents changes in task set preparedness. The term (*τ* + (1 − *τ*)*L*(*t*_*j*_)) determines the relationship between task set preparedness and forgetting. The term *L*(*t*_*j*_) represents the context loss that varies as a function of elapsed time *t*_*j*_. Larger context losses have a bigger impact on task set preparedness. We define the context loss function *L*(*t*) as follows:

5 $$L(t)=1-{e}^{-\gamma t}$$

**Fig. 4:**
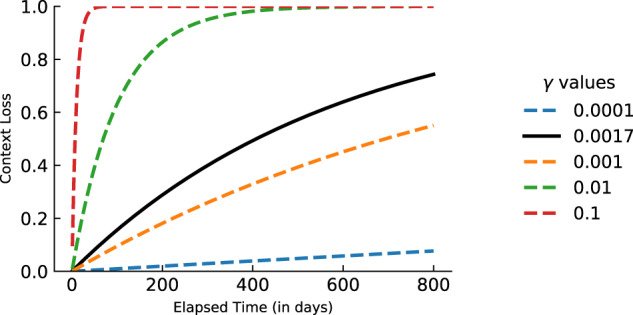
Loss of context *L* as a function of time delay *t*_*j*_. Loss of context as a function of the time delay (*t*_*j*_) between game session *j* and *j* − 1 for different parameter settings of *γ*. Colours represent different values of *γ*. The black curve shows the estimated context loss function for the Lumosity data.

This function can capture a wide variety of temporal patterns describing context loss during the retention interval. Figure 4 shows the context loss for different values of *γ*. This functional form of loss allows for a variety of forgetting functions. When *γ* = 0 there is no context loss; as *γ* increases the loss becomes more rapid. For both games in our analysis, the best-fitting context loss function corresponds to the black line. For this function, 50% of the context is lost after a delay of about 450 days and close to 80% is lost after 800 days. Similar to *M*_3_, we assume *γ* to be shared across participants.

In the analysis that follows, we seek empirical aspects of real-world learning within the Lumosity data set that adjudicate between these four theoretical approaches, and provide fits of the competing models to the data in the Lumosity data set we introduce in the next section.

## Results

The Lumosity platform provides a number of games that tap memory, attention, flexibility, speeded processing, and problem solving. In the Lumosity program, users are given a recommended daily training session of five different cognitive training games. One five-game session takes ~15 min to complete. Outside of the training sessions, Lumosity users can also opt to select and play games directly from the library of available games. We describe in the *Methods* section the data and data processing steps we took for the analyses presented here.

### The effect of gaps in practice on retention and relearning

Our primary objective is to provide a means of understanding the course of skill learning over extended time periods when interruptions to practice are common, idiosyncratic, and only partially regular. The learning curves shown in Fig. 1 reveal a distinctive saw-toothed pattern, in which each new session of practice begins at a level of performance that is lower than the last level of performance but quickly rises. This effect is so dramatic that, even at a delay of almost 1 year (bottom row), and a considerable reduction in performance, performance recovers after only a few gameplays in the session.

Because this effect on relearning is one of the most distinctive features of long-timescale learning, and because it holds considerable promise in adjudicating between the theoretical positions outlined here, we provide a more in-depth analysis of this result.

**Fig. 5:**
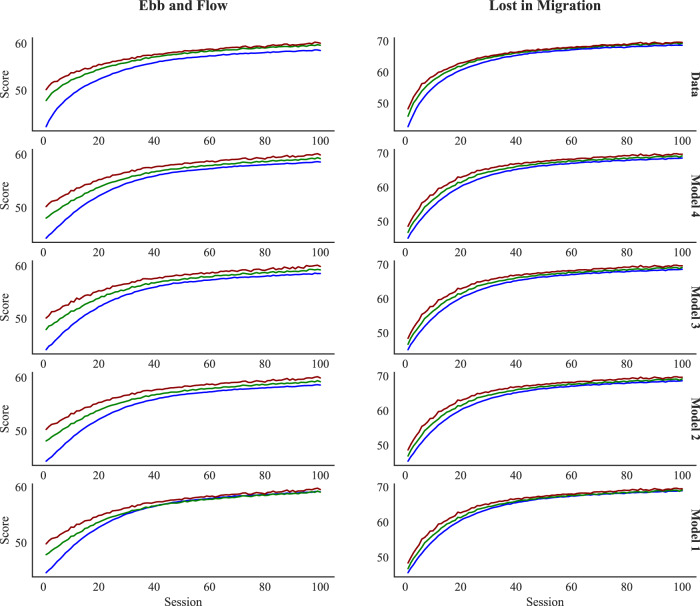
Observed and predicted aggregate learning curves over sessions for the 1st to 3rd gameplays within each session for Ebb and flow and lost in migration. The performance score (vertical axis) is assessed by the number of correct decisions per game play.

Figure 5, top row, provides a unique perspective on learning and relearning effects. Each curve represents a learning function conditionalized on the specific gameplay within each session. So, for example, the blue curve represents performance on the very first gameplay of each session, across many sessions. Learning across sessions is ample and mirrors traditional learning curves. But, importantly, each consecutive gameplay reveals shallower growth over sessions, revealing a new angle from which to view the effects of warm-up decrements and recovery.

**Fig. 6:**
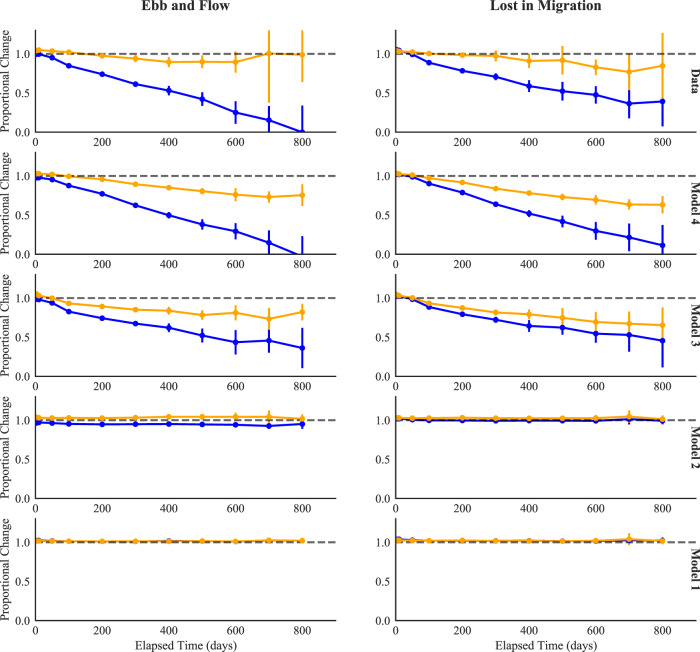
Retention of performance as a function of delay between sessions. Results are shown for Lost in Migration and Ebb and Flow. Retention is assessed by the proportional change in performance relative to baseline. A value of one (dashed line) indicates no performance loss relative to the last gameplay in the previous session. Results are separated by the first and second gameplay in the session after the retention interval. The error bars are the 95% confidence intervals of the mean.

A related phenomenon is shown in Fig. 6, top row, which plots performance on the first and second gameplays as a function of the retention interval from the last practice session. A novel and appealing feature of this analysis is the extreme range in intervals, which range from only a few days to >2 years. Change is measured proportionally with respect to the first gameplay in the previous session (prior to the delay). For the first game within the session, delay has a substantial effect on the proportional change in performance: a delay of 100 days leads to a 20% reduction in performance and a delay of 400 days leads to a 50% reduction. The dramatic finding here is that those losses are quickly offset by a very small amount of practice. This effect can be seen in the forgetting function for the second gameplay within the session, which is flat or nearly so. In the *Lost in Migration* game, in which a loss of nearly 100% of prior performance is apparent after 800 days, a single gameplay restores performance to the same level that was achieved in the prior practice session. Relearning benefits of this magnitude and over these time scales have not previously been documented, to our knowledge. They provide a strong test of the computational approaches to learning.

### Computational models of practice

We fit the four models *M*_1_, *M*_2_, *M*_3_, and *M*_4_ to the data from each individual user. In these fits, some model parameters are unique to each individual and some model parameters are shared among all users (see Methods for details). We evaluate the models in two different ways. First, we assess the ability of the models to capture the qualitative trends in Figs. 5 and 6. Second, we use predictive evaluation methods to assess the ability of the models to predict unseen (“out-of-sample”) data^32^. Specifically, we assessed out-of-sample model fit by the root mean squared error (RMSE; lower values indicate better predictive performance), for all four models: 80% of the data was used for model estimation and 20% of the data was held out as a test set to assess the ability of the models to predict performance on new (out-of-sample) data. In order to highlight the distinct contributions of the four models, we report the predictive model fit for all held-out data, and also across subsets of the held-out data that differ in retention interval, varying from quite short (< 1 h, corresponding to within-session gameplay delays) to very long (>100 days). These partitions were determined ahead of time based on data availability and were decided upon prior to viewing the outcomes of the analysis.

Table 1 shows the out-of-sample model results (See Supplementary Table 1 for in-sample results). Note first that both the single-parameter learning model (*M*_1_) and the traditional two-timescale model (*M*_2_) are not competitive in accounting for learning in this data set. Yet *M*_2_ provides a substantial, across-the-board improvement over *M*_1_. This outcome reveals the ubiquity and substantial nature of warm-up decrements in naturalistic learning.

**Table 1:** Predictive performance of the models assessed by root mean squared error (RMSE) on out-of-sample data. Numbers in parentheses show number of participants in the evaluation. Best performing models for each game and data subset are highlighted in bold.

	Between gameplay delay (*t*)				
	All Data	*t* < 1 h	*t* < 10 days	*t* < 100 days	*t* > 100 days
Game 1: lost in migration	(19463)	(19463)	(19463)	(18292)	(4680)
*M*_1_: baseline learning	3.61	3.68	3.36	3.77	5.90
*M*_2_: two-timescale learning	3.56	3.65	3.31	3.59	5.41
*M*_3_: two-timescale learning and forgetting	3.56	3.66	**3.30**	**3.54**	**4.72**
*M*_4_: interactive	**3.55**	**3.64**	**3.30**	3.55	5.17
Game 2: ebb and flow	(19694)	(19694)	(19694)	(17845)	(3336)
*M*_1_: baseline learning	4.56	4.58	4.33	4.99	9.26
*M*_2_: two-timescale learning	4.54	**4.57**	4.33	4.90	8.94
*M*_3_: two-timescale learning and forgetting	**4.53**	4.59	**4.31**	**4.79**	**7.61**
*M*_4_: interactive	**4.53**	4.58	4.32	4.81	8.17

Numbers in parentheses show number of participants in the evaluation. Best performing models for each game and data subset are highlighted in bold.

For lost in migration, M_4_ provides the most accurate predictions overall. For ebb and flow, models M_3_ and M_4_ provide equivalently accurate predictions. Taken together, M_4_, which uniquely allows for a degree of interactivity between the two timescales of learning, provides the most general account of performance. This outcome is particularly compelling in light of the fact that the much less flexible M_3_ outperforms the interactive model at six of the eight interval subsets. That is, a model that allows for two timescales of learning and independent forgetting can accurately describe data that arise from a limited range of intervals between practice sessions, but only the interactive model can account for the patterns of learning, relearning, and forgetting over the wide range of intervals produced by naturalistic learning in this set of games. This outcome provides a salutary example of the dangers of generalizing models developed from laboratory tasks to real-world data.

The implications of the interactivity built into model *M*_4_ can be clearly viewed in the second row of Figs. 5 and 6. In those plots, the predictions of *M*_4_ for relearning are plotted in the same manner as the empirical data in the top row. Model *M*_4_ accurately accounts for the steeper growth for earlier than later gameplays (Fig. 5) and the rapid recovery from a single gameplay, as revealed by the much shallower forgetting function evident on the second than the first gameplay (Fig. 6). The relearning predictions of the other models do not capture this pattern.

## Discussion

The behaviour of learners who guide their own practice over extended periods reveals much about the process of learning. It is a testament to the deep historical investment made by researchers in the learning and cognitive sciences that all of the important aspects of a robust model of naturalistic learning were anticipated by researchers using smaller datasets in which practice was much more constrained.

There is ample prior research on schedules of learning, even over extreme durations (e.g.^33–35^), but no research (to our knowledge) that combines these characteristics with the opportunity for learners to control their own scheduling of practice. Because those circumstances are common in real-world learning, we believe that datasets like the one we have made use of here can be extremely valuable in helping generalize findings from the lab and the classroom into the wild and wooly conditions of self-guided study. We make our data publicly available to facilitate more work in this area. The key concepts at the core of our findings are as follows.

First, learning proceeds over multiple timescales. It is apparent that two components of learning underlie performance in the tasks under investigation here. We have suggested that these two components are *skill*—the fundamental knowledge relevant to completing the task—and *task set*—the body of knowledge relevant to translating that skill into performance. Consider the popular game Tetris, in which a player must rotate objects as they descend in order to place them strategically within a growing collection of such objects. As a player practices this game, they will get better—they will score more points and make their chosen moves faster and with greater accuracy. Some of what is learned in the course of practice is procedural skill that is acquired steadily but *slowly* through actual hands-on practice. Other aspects of performance are linked to the task-relevant set of knowledge that presents a bottleneck for revealing that skill, like knowing the keypresses that correspond to different manoeuvres, remembering to check the visual signal that previews the upcoming piece, and having a long-term strategy for maximizing point gains. Task-set knowledge can be learned and expressed declaratively (though it may eventually become automated and part of the skill itself; ^20^). Most importantly, these elements of performance can be partially or wholly forgotten between practice sessions, but can also be quickly reacquired within each session and so may be aptly described as the “fast” component of learning. In contrast, skill, as implemented in the models here, is permanent and not susceptible for forgetting.

The notion of two timescales for learning has important historical precedents. One perspective on “fast” learning emphasizes the role of *reminiscence*, through which fluctuations in the conditions of performance lead to an increasing number of skill-relevant memories becoming available over time^36,37^. Such theories provide little illumination on the core issue presented here, however: the juxtaposition of rapid learning within a session with slow learning across sessions. Consolidation over periods of rest has also been postulated to play a role in promoting long-term performance^38^, but such theories run aground on the finding that interpolated tasks that interfere with consolidation actually increase, rather than decrease, long-term performance^39^.

An insightful perspective is provided by refs. ^40,41^, who distinguished between short-term gains in performance that accrue within a session and long-term gains that can only arise when gaps between learning sessions provide spaced opportunities to retain knowledge. This theory was supported by a later reanalysis of Snoddy’s data^42^ and spurred the development of a more complex theory that maintained the critical distinction between long-term learning and short-term effects^14^. That distinction is embodied in nearly all of the models we have considered here and is critical to predicting performance over either limited retention intervals (in the form of Model *M*_3_) or widely varying retention intervals (in the form of Model *M*_4_). Given this conceptualization of task set, it may be profitable to look to research on task-switching effects as a starting point for understanding the nature of “fast” learning^43^.

Another important concept that constrained model development is that forgetting interleaves learning events. Historical perspectives on learning pay great heed to the practice or study events that enhance performance but mostly ignore the occasions for forgetting that arise between those event. Opportunities for forgetting are limited in laboratory tasks in which learning events are scheduled back-to-back with little rest, but are ample in real-world learning, where practice must be co-scheduled with other tasks and is subject to constraints and interruptions.

A two-timescale model of learning possesses a rudimentary form of forgetting, insofar as within-session learning is presumed to be temporary and is thus “forgotten” between practice study sessions. We see in the data presented here that this view is an incomplete means of accounting for the fullness of long-term learning with extensive and varied breaks in practice. Performance drops more substantially after a long than a short break. This finding is intuitive but it is important to note the extent to which forgetting grows after even exceptionally long breaks. Performance drops as much between 700 and 800 days of time off as it does between 0 and 100 days off (see Fig. 6). This outcome is inconsistent with the concept of task-set learning being “fast” and temporary; the outcomes of our model-fitting endeavour support the view that an additional explicit means of accounting for variable forgetting (as embodied in Model *M*_3_) provides a substantial improvement over the traditional two-timescale model (*M*_2_).

Finally, our results show the components of learning are interactive. The relearning data considered here indicate that within-session learning is more effective when skill is greater. This is revealed by the exceptionally rapid recovery of prior performance levels, and can only be accounted for with a model that explicitly allows for interactivity between these components.

This interactivity is evident in models of learning suggested by refs. ^21–24^. We have provided an alternative here that is designed to be robust to the exceptionally long durations present in the Lumosity dataset, and provide a more in-depth application of one prominent model (the Predictive Performance Equation^21,24^) to these data in the Supplemental Materials.

There is much to be gained from the application of theories and computational models of learning to real-world data, even as we must deal with the complexities that they bring with them. As mainstream learning science has moved from the conditions of extreme control provided by animal-learning paradigms to the use of humans in laboratories and classrooms, we must now consider what can be gained from allowing the learner to re-enter their most natural milieu—learning on their own time, at their own schedule.

## Methods

Our analysis is based on an analysis of a data set from the Lumosity cognitive training platform. Because this project involves a retrospective analysis of existing data, the research is exempt from IRB review as determined by the University of California Irvine IRB.

**Fig. 7:**
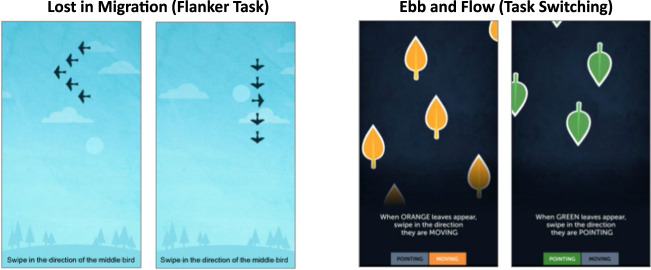
Screenshots of the two cognitive games in this research. Lost in Migration (flanker task) and Ebb and Flow (task switching).

The platform provides a number of different games for users that are intended to tap memory, attention, flexibility, speeded processing, and problem solving. Millions of people play these games, providing a very rich platform on which to study learning. Users access the platform through a browser or app but the data set for this study is based on users who primarily used the web-based version of the games. The data set comprised the gameplay event history for two cognitive games, a flanker task (“Lost in Migration”) and a task switching game (“Ebb and Flow”). This data set includes 194,695 users, 389,389 individual learning curves, and 41,006,715 single gameplay events. Our analysis is based on a sub-sample of 19,463 users in the Flanker task and 19,694 users in the Task Switching game. This data set is available online (see data availability statement). Figure 7 shows screenshots of two games we use for our analysis.

### Flanker Task

This selective attention game is inspired by the Eriksen flanker task^44^. The goal is to respond to the direction of the target (a bird) and ignore the direction of distractors that flank the target. During each trial, the target and distractors are arranged in different spatial layouts. Players use the arrow keys to indicate which direction the target is pointing; the layout and orientation of the distractors varies from trial to trial.

### Task Switching

This is a game designed to test the ability to switch between different tasks^45^. Users have to shift focus between two different rules depending on the colour of the leaves. When the leaves are green, the user has to determine the direction in which the leaves are pointing and respond accordingly. When the leaves are orange, the user has to respond based on the direction that they are moving.

### Performance assessments

Each gameplay event has a fixed duration: 45 s for the flanker task and 60 s for task switching. At the end of each gameplay event, users are provided feedback on mean response time per trial, mean accuracy, and a score that is based on the total number of correct trials completed within the fixed time period as well as bonus points based on a variety of factors (e.g., streaks of correct responses). The total score is the focal point on the feedback screen, so it can be assumed that the conditions foster a combination of speed and accuracy.

### Participants

We worked with a subset of the data that includes 194,695 users. Basic demographic information is available from the aggregate statistics of users of Lumosity. The majority of users are female (59%), with 36% males and 5% of users who did not provide gender information. We coded the age of users in 3 bins leading to the following breakdown of the user sample: 21-40 (18.3%), 41-60 (41.8%), and 61-80 (40.0%). The youngest age group (1–20) is omitted from all analyses because of the relatively small sample size and the heterogeneous nature of this age group. Most users live in the United States (63%), with substantial populations from Canada (9.6%), Australia (9.1%) and Great Britain (2.2%). Consequently, it is a sample heavily biased towards Western nations.

### Data processing

The raw data is described at the individual trial level (i.e., individual decisions within a particular gameplay event) and include response time, accuracy, as well as the type of condition associated with the trial. In the raw data, any trial with a response time >5 s was coded as 5 s. For the purpose of this research, we analyzed the data summarized at the gameplay event level. Specifically, we focused on the *number of correct trials completed per game play*, a value that is closely related to the inverse of the mean response time for correct decisions. It is also closely related to the point score received by the user, but it is not identical because we omitted bonus points that are part of the game scoring.

The data set contains the full gameplay history across the two games spanning a period from December 18, 2012 to October 31, 2017. Users spent a mean of 2.5 years on the platform. The flanker task and task switching game were played a median of 86 and 81 times, respectively. Some of the gameplays had timestamps but lacked any recorded gameplay data. We removed these missing records from the data sets.

#### Clustering gameplays into sessions

The user activities on the Lumosity platform can be grouped into sessions on the basis of the elapsed time between gameplays. When users replay, they play either immediately (roughly within 15 s after the last game ends) or wait for several hours but more typically at least 1 day. We define a session here as all consecutive gameplays for which there is no between-gameplay break of >1 h. Therefore, any consecutive gameplays with a delay of 1 h or longer are defined to be part of different sessions. Based on this definition, the 41,006,715 gameplays were grouped into 34,722,958 total sessions. The majority of sessions (81%) include only a single gameplay. When individuals played multiple times, shorter sessions were more likely than longer sessions (9.7% sessions with 2 gameplays, 3.9% sessions with 3 gameplays, 1.8% sessions with 4 gameplays).

#### Creating a subsample of users

Instances of multiple gameplays within a session are crucial to investigating relearning. Hence, we restricted our sample to participants with a ratio of total gameplays to total sessions >1.5. This constrained the size of our data to 53,745 and 48,330 participants in the two games. From this subset, we randomly sampled 25,000 participants for each game. We further restricted the data to only include participants that had >50 game sessions. The final subset consisted of learning curves of 19,463 users that played Lost in Migration and 19,694 users who played Ebb and Flow.

In our final subset, 8,330,182 gameplays (88% of total gameplays) were played after delays of <7 days. Long delays between sessions also occur: 32,436 were spaced 1 month apart and 667 sessions were spaced an entire year apart. Figure 2b shows the spread of elapsed time between consecutive sessions in the subsample of our two games. Supplementary Table 3 shows additional descriptive statistics of the subsample.

For both games, to split the data into train and test sets, we follow a conditional sampling approach. We create two subsets of the data, one with elapsed times between game sessions >100 days and the other with elapsed times less than a 100 days. From each subset, we hold out as test set 20% of the gameplays. The rest of the data was used as training data. This allows us to ensure the presence of trials with large delays in both the train and test data.

#### Estimating proportional performance changes

Because our analysis highlights performance losses or gains over sessions and over individuals that vary in overall performance, it is convenient to scale any performance changes relative to baseline performance by assessing the *proportional* change in performance. We use this metric for the results shown in Fig. 6. To illustrate this metric, suppose an individual performed at levels *X* and *Y* for the *t* and *t* + 1 gameplay. Suppose further that the individual started at the beginning of practice at baseline level *B*. The proportional change from gameplay *t* to gameplay *t* + 1 is then evaluated as (*Y* − *B*)/(*X* − *B*). For example, a value of of 0.7 indicates a loss of 30% of the performance improvements (relative to baseline) between the *t* and *t* + 1 gameplay. Values smaller than one indicate a loss in performance whereas values larger than one indicate a gain. To perform these calculations, an estimate of the baseline performance (*B*) at the start of practice is needed. We use the average scores of the observed performances on the first 3 gameplays as an estimate of baseline performance. In order to avoid instabilities due to small values in the denominator (*X* − *B*), we excluded observations in our analysis in which (*X* − *B*) < 2 (this affected <2% of scores). Therefore, we are excluding observations where little or no progress was made relative to the start of practice.

### Model estimation

Model parameters were estimated using a maximum-likelihood procedure, with the objective function defined as the root-mean-square error between the true game score and the game score predicted by the model. We implemented automatic differentiation to numerically calculate the gradients and used the Adam algorithm^46^ to carry out adaptive gradient descent to learn the parameter values. Estimation can be challenging if appropriate constraints are not placed on model parameters. We applied the following constraints on parameter values. The learning rate parameter (*λ*) in each model was restricted to values between 0 and 0.1. Other (*τ*, *β*, and *γ*) were restricted to values between 0 and 1.

Each participant had their own set of parameters in all models with the exception of parameter *γ* which was shared across participants in models *M*_3_ and *M*_4_. For *M*_3_ and *M*_4_, we first computed the maximum-likelihood estimates of the *γ* parameter using a subset of 9463 and 9694 participants’ data for the two games. For the rest of the 10,000 participants in both games, we used the *γ* parameter estimated from the subset of participants in the previous step.

Supplementary Table 2 shows the estimated parameter values from the application of the four models to the Lumosity data. Supplementary Fig. 1 shows the distribution of estimated parameter values across users. Supplementary Fig. 2 shows the model fit for Model *M*_4_ for part of the learning trajectories of a few illustrative users.

### Predictive performance equation

The predictive performance equation (PPE) is a model of the spacing effect that aims to describe the dynamics of human learning and forgetting^24^. In this section, we explain how we applied the PPE model to the Lumosity data set. We follow notation and follow predefined parameter settings as specified by ref. ^24^.

In PPE, the activation *M* of item *n* is a function of elapsed time *T* (in seconds) and the number of practice trials *N*:

6 $${M}_{n}={N}^{c}{T}^{-d}$$

where *c* is the learning rate, which is set to 0.1^24^ and *d* is the decay rate. *d* is calculated as a function of elapsed times between successive practice opportunities (*lag*_*j*_):

7 $${d}_{n}=b+m\left(\frac{1}{n-1}\mathop{\sum }\limits_{j=1}^{n-1}\frac{1}{ln(la{g}_{j}+e)}\right)$$

The value of decay rate approaches the decay intercept parameter *b* when lags are long and approaches *b* + *m* when the lags are short (where *m* is the decay slope parameter). Elapsed time *T* is calculated as the weighted sum of the time since each previous practice event:

8 $${T}_{n}=\mathop{\sum }\limits_{i=1}^{n}{w}_{i}{t}_{i}$$

where *w*_*i*_ is the weight assigned to each event *i*:

9 $${w}_{i}=\frac{{t}_{i}^{-x}}{\mathop{\sum }\nolimits_{j = 1}^{n}{t}_{j}^{-x}}$$

The variable *x* modulates the weighting of practice events and is set to 0.6. The PPE further maps activation *M*_*n*_ to performance *P*_*n*_ via a logistic function:

10 $${P}_{n}=\frac{1}{1+\exp \left(\frac{\tau -{M}_{n}}{s}\right)}$$

The parameters *τ* and *s* control the slope and intercept of the logistic function, respectively.

To adapt the PPE to the context of the Lumosity gameplay data, we treat each gameplay (*j*) as a practice event and use activation *M*_*j*_ to estimate the score in that gameplay via the following mapping:

11 $${y}_{j}=A+U{M}_{j}^{k}$$

where *A*, *U* and *k* are player-specific scaling parameters. Note that we have simulated a number of alternative functional mappings and all yielded similar results.

We fit the PPE separately to each participant’s gameplay history and used a maximum likelihood estimation procedure to estimate the model’s parameters. The objective function was defined as the mean square error between true game score and game score predicted by the PPE. We inferred *A*, *U*, and *k* parameters for every individual participant and decay parameters *b* and *m* shared across participants. We implemented automatic differentiation to numerically calculate the gradients and used the Adam algorithm to carry out adaptive gradient descent to learn the parameter values. Consistent with the procedures followed by Walsh et al., *b* and *m* parameters were restricted to values between 0 and .2.

The resulting predictive performance of the PPE model, along with the four models described in the main paper is shown in Supplementary Table 1. Supplementary Figs. 3 and 4 show the aggregate model predictions for within and between sessions learning as well as the retention as a function of delay.

## Supplementary information

Supplementary Material

## Data Availability

Preprocessed versions of the data are available at: https://osf.io/zkyr8/?view_only=cb500b45c76f448ea486dd0ec2e6ea4a.

## Code availability

The code used to analyze the data and generate the images used in this paper is available at: https://osf.io/zkyr8/?view_only=cb500b45c76f448ea486dd0ec2e6ea4a.
