# Supplementary material for: Comparing models of learning and relearning in large-scale cognitive training data sets
Source: NPJ Sci Learn. 2022 Oct 4;7:24. doi: 10.1038/s41539-022-00142-x (PMC9532425; doi:10.1038/s41539-022-00142-x)
Supplement: Supplementary file 1 — Supplementary Material [file 41539_2022_142_MOESM1_ESM.pdf]

Supplement: Comparing models of learning and relearning in large-scale cognitive training data sets

Aakriti Kumar[1], Aaron S. Benjamin[2],
Andrew Heathcote[3], and Mark Steyvers[1]

[1]University of California, Irvine
[2]University of Illinois at Urbana-Champaign
[3]University of Newcastle, Australia

Supplementary Tables

Supplementary Table 1: Predictive performance of the models assessed by root mean squared error on in-sample and out-of-sample data. Numbers in parentheses show number of participants in the evaluation. Best performing models for each game and data subset are highlighted in bold.

		Between Gameplay Delay (t)			
	All Data	$t < 1$ hour	$t < 10$ days	$t < 100$ days	$t > 100$ days
**OUT-OF-SAMPLE DATA**					
Game 1: Lost in Migration	(19463)	(19463)	(19463)	(18292)	(4680)
M_1: Baseline learning	3.61	3.68	3.36	3.77	5.90
M_2: Two-timescale learning	3.56	3.65	3.31	3.59	5.41
M_3: Two-timescale learning and forgetting	3.56	3.66	**3.30**	**3.54**	**4.72**
M_4: Interactive	**3.55**	**3.64**	**3.30**	3.55	5.17
PPE: Predictive Performance Equation	3.73	3.80	3.52	3.73	5.43
Game 2: Ebb and Flow	(19694)	(19694)	(19694)	(17845)	(3336)
M_1: Baseline learning	4.56	4.58	4.33	4.99	9.26
M_2: Two-timescale learning	4.54	**4.57**	4.33	4.90	8.94
M_3: Two-timescale learning and forgetting	**4.53**	4.59	**4.31**	**4.79**	**7.61**
M_4: Interactive	**4.53**	4.58	4.32	4.81	8.17
PPE: Predictive Performance Equation	5.16	5.11	5.12	5.17	9.13
**IN-SAMPLE DATA**					
Game 1: Lost in Migration	(19463)	(19463)	(19463)	(18292)	(4680)
M_1: Baseline learning	3.56	3.89	3.44	3.55	5.03
M_2: Two-timescale learning	3.49	3.84	3.38	3.45	4.79
M_3: Two-timescale learning and forgetting	**3.46**	3.84	**3.37**	**3.36**	**3.40**
M_4: Interactive	3.47	**3.83**	3.37	3.42	3.87
Game 2: Ebb and Flow	(19694)	(19694)	(19694)	(17845)	(3336)
M_1: Baseline learning	4.46	4.80	4.27	4.76	7.43
M_2: Two-timescale learning	4.43	4.76	4.25	4.69	7.25
M_3: Two-timescale learning and forgetting	**4.36**	**4.77**	**4.23**	**4.54**	**4.91**
M_4: Interactive	4.39	4.78	4.24	4.62	5.49

Supplementary Table 2: Means of individual specific parameters in models M_1, M_2, M_3, M_4 based on 19,463 participants in game 1 and 19,696 participants in game 2: Numbers in parentheses show the 5 and 95 quantile estimates of the parameter estimates

Model	A	U	λ	τ	β	δ
Game 1: Lost in Migration						
M_1	61.49 (45.71,79.12)	19.53 (4.03, 35.33)	0.036 (0.002, 0.099)	-	-	-
M_2	63.83 (47.88, 82.71)	17.53 (3.81, 34.09)	0.035 (0.002, 0.099)	0.21 (2e-5, 0.97)	0.66 (4e-6, 0.99)	-
M_3	60.25 (44.51, 79.68)	18.56 (4.49, 33.82)	0.039 (0.002, 0.099)	0.19 (2e-3, 0.92)	0.63 (8e-7, 0.99)	0.25 (4e-6, 0.99)
M_4	63.83 (48.14, 82.34))	17.54 (4.31, 31.69)	0.035 (0.003, 0.099)	0.21 (8e-6, 0.97)	0.65 (8e-7, 0.99)	-
Game 2: Ebb and Flow						
M_1	71.84 (54.23, 85.41)	29.46 (13.09, 46.79)	0.039 (0.004, 0.099)	-	-	-
M_2	73.49 (59.56, 89.22)	28.16 (12.65, 45.75)	0.039 (0.005, 0.099)	0.12 (4e-5, 0.75)	0.50 (4e-6, 0.99)	-
M_3	70.10 (53.33, 87.61)	28.69 (12.92, 45.34)	0.037 (0.004, 0.099)	0.17 (7e-5, 0.85)	0.32 (2e-7, 0.99)	0.22 (2e-6, 0.77)
M_4	76.51 (57.40, 101.38)	27.77 (12.88, 45.18)	0.037(0.005, 0.099)	0.19 (1e-6, 0.99)	0.50 (8e-8, 0.99)	-

Supplementary Table 3: Descriptive statistics of the Lumosity sample

Descriptive Statistics	Lost in Migration	Ebb and Flow
Elapsed time between sessions in days (mean)	8.18	7.72
Elapsed time between sessions in days (median)	2.11	2.18
Longest retention interval per user in days (mean)	159.61	152.89
Longest retention interval per user in days (median)	114.4	105.27
Number of gameplays (mean)	400.8	299.32
Number of gameplays (median)	254	223

Supplementary Figures

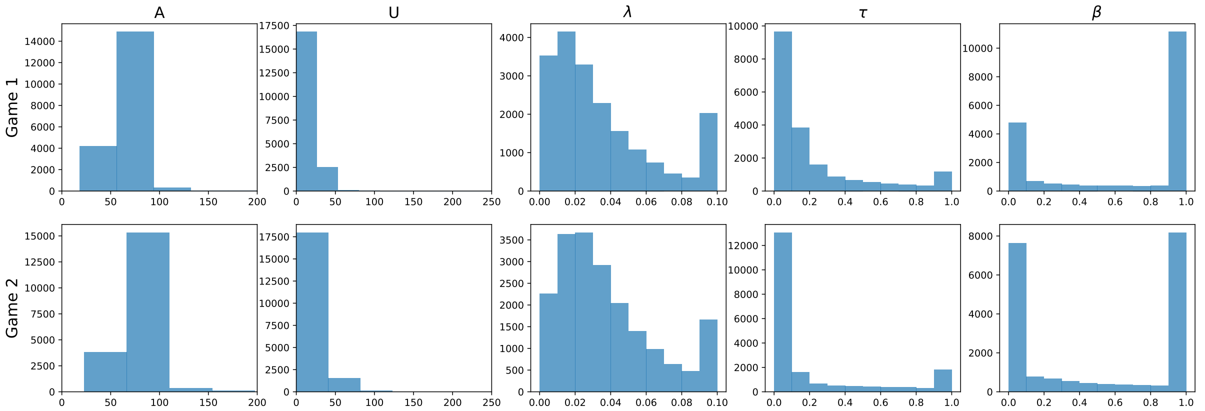

Supplementary Figure 1: Histograms of the estimated random parameters of model M_4 for Game 1 and Game 2

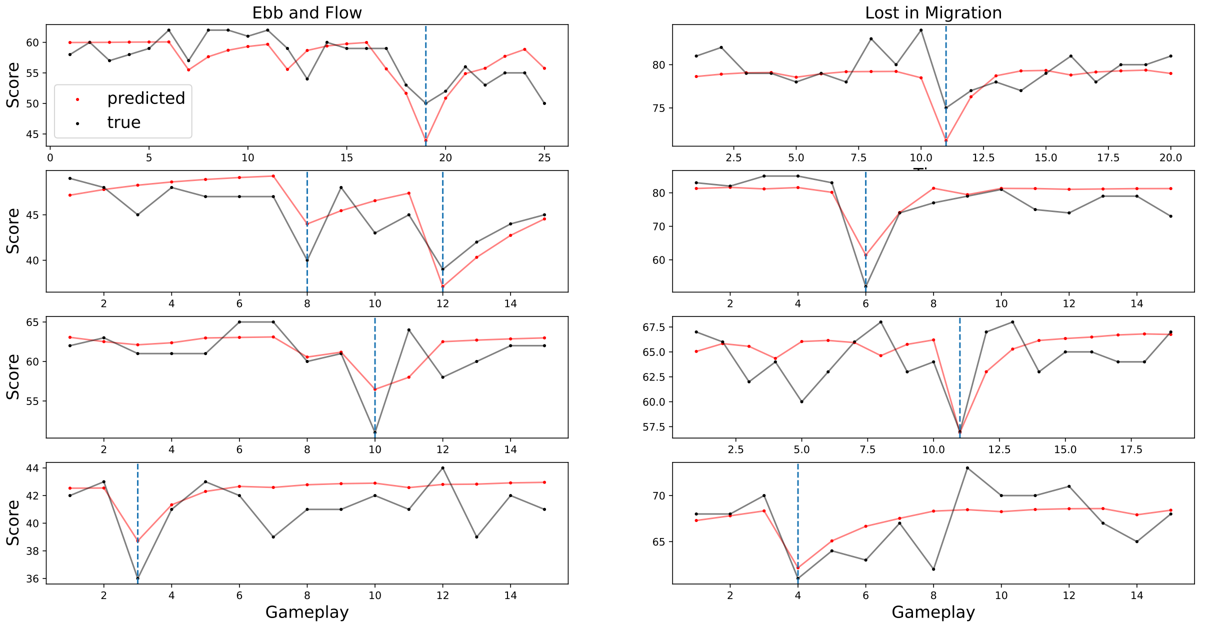

Supplementary Figure 2: Model M_4's predictions and true scores for a subset of gameplays of 4 participants in Game 1: Ebb and Flow and Game 2: Lost in Migration. The blue dashed lines correspond to gameplays which had a retention interval of at least 100 days.

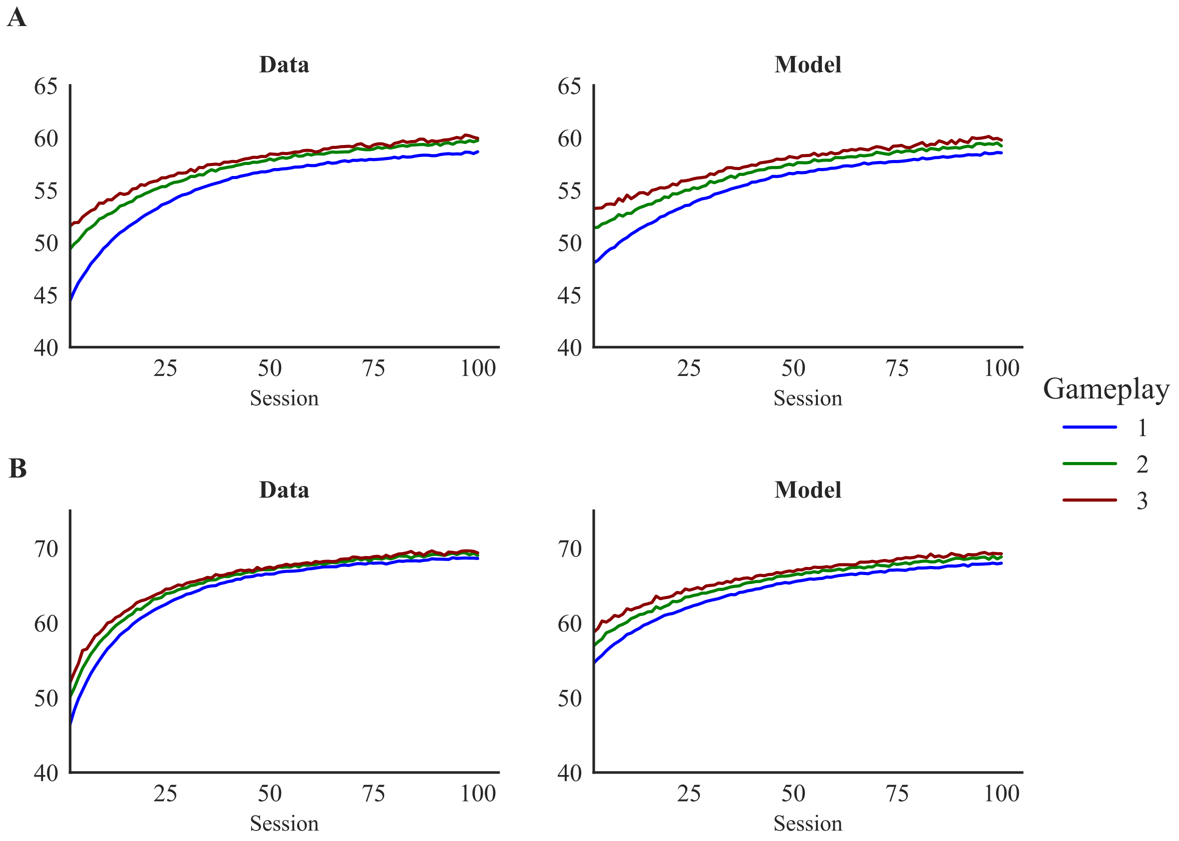

Supplementary Figure 3: **Observed and predicted aggregate learning curves over sessions for the 1st to 3rd gameplays within each session**. The results are separated by the games (A) Lost in Migration and (B) Ebb and Flow. Performance is assessed by the number of correct decisions per game play. Model predictions are based on the PPE model.

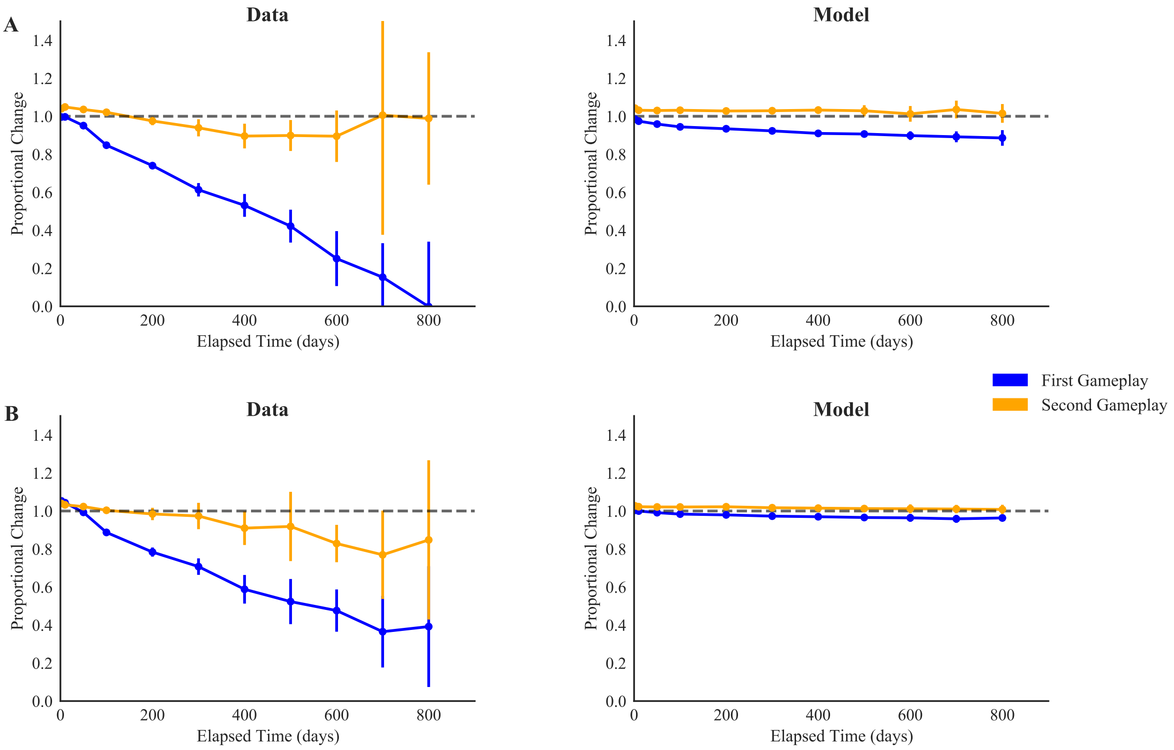

Supplementary Figure 4: **Retention of performance as a function of delay between sessions for the PPE model**. The results are shown for the games (A) Lost in Migration and (B) Ebb and Flow. Retention is assessed by the proportional change in performance relative to baseline. A value of one (dashed line) indicates no performance loss relative to the last gameplay in the previous session. Results are separated by the first and second gameplay in the session after the retention interval. Model predictions are based on the PPE model. While the PPE model is in principle capable of capturing effects of delay on learning and retention, the results show that it does not quantitatively capture both short term and long term delay effects at the same time in the Lumosity game play data. However, it is important to keep in mind the extreme range of delays in our data - ranging from the order of minutes between consecutive gameplays to the order of years between (some) consecutive sessions. One possibility is that the PPE model will have to be extended or modified to handle the wide range of time scales underlying the Lumosity data.
